# Do images of a personalised future body shape help with weight loss? A randomised controlled study

**DOI:** 10.1186/s13063-017-1907-6

**Published:** 2017-04-18

**Authors:** Gemma Ossolinski, Moyez Jiwa, Alexandra McManus, Richard Parsons

**Affiliations:** 1 Department of Medical Education, Curtin University, Perth, WA 3845 Australia; 2 Melbourne Clinical School, School of Medicine Sydney, The University of Notre Dame Australia, Werribee, VIC Australia; 3 Faculty of Health Sciences, Curtin University, Perth, WA Australia; 4 School of Occupational Therapy and Social Work and School of Pharmacy, Curtin University, Perth, WA Australia

**Keywords:** Weight loss, Medical informatics applications, Obesity, Intervention study, Health promotion

## Abstract

**Background:**

This randomised controlled study evaluated a computer-generated future self-image as a personalised, visual motivational tool for weight loss in adults.

**Methods:**

One hundred and forty-five people (age 18–79 years) with a Body Mass Index (BMI) of at least 25 kg/m^2^ were randomised to receive a hard copy future self-image at recruitment (early image) or after 8 weeks (delayed image). Participants received general healthy lifestyle information at recruitment and were weighed at 4-weekly intervals for 24 weeks. The image was created using an iPad app called ‘Future Me’. A second randomisation at 16 weeks allocated either an additional future self-image or no additional image.

**Results:**

Seventy-four participants were allocated to receive their image at commencement, and 71 to the delayed-image group. Regarding to weight loss, the delayed-image group did consistently better in all analyses. Twenty-four recruits were deemed non-starters, comprising 15 (21%) in the delayed-image group and 9 (12%) in the early-image group (χ^2^(1) = 2.1, *p* = 0.15). At 24 weeks there was a significant change in weight overall (*p* < 0.0001), and a difference in rate of change between groups (delayed-image group: −0.60 kg, early-image group: −0.42 kg, *p* = 0.01). Men lost weight faster than women. The group into which participants were allocated at week 16 (second image or not) appeared not to influence the outcome (*p* = 0.31). Analysis of all completers and withdrawals showed a strong trend over time (*p* < 0.0001), and a difference in rate of change between groups (delayed-image: −0.50 kg, early-image: −0.27 kg, *p* = 0.0008).

**Conclusion:**

One in five participants in the delayed-image group completing the 24-week intervention achieved a clinically significant weight loss, having received only future self-images and general lifestyle advice. Timing the provision of future self-images appears to be significant, and promising for future research to clarify their efficacy.

**Trial Registration:**

Australian Clinical Trials Registry, identifier: ACTRN12613000883718. Registered on 8 August 2013.

**Electronic supplementary material:**

The online version of this article (doi:10.1186/s13063-017-1907-6) contains supplementary material, which is available to authorized users.

## Background

The prevalence of obesity in developed regions has risen at an alarming rate over the past 30 years, and continues to rise despite public awareness of the associated risk of chronic disease [1]. Outside of surgical intervention, weight loss for the individual relies on lifestyle change that promotes an overall reduction of dietary caloric intake and an increase in physical energy expenditure. Being aware of the need to lose weight is usually not enough to produce sustained lifestyle change. The individual requires the initial motivation to change unhealthy dietary habits and to revise entrenched sedentary behaviours, followed by continued effort to sustain the healthy lifestyle choices.

Health professionals are often counselled for weight loss, either in response to a direct request from an individual or because of an associated illness that requires medical attention. Weight-loss counselling in the general practice setting often takes place in time-constrained consultations [2, 3]. In addition, a lack of patient motivation and failure to adhere to recommendations are often cited as a barrier to effective weight-loss intervention [4].

New technology, in the form of digital self-representations, known as Avatars, have been shown to improve health-related behaviours, including diet and exercise choices [5, 6]. Written advice that is tailored and personalised for the individual has been shown to be more effective than traditional collective advice for diet and exercise behaviour change [7, 8]. Empirical evidence from a pilot study published in 2015 suggests that self-representations in the form of computerised future self-images could enhance weight loss in women who are trying to lose weight [9]. This is consistent with the theory that a fundamental human need is to forge or maintain bonds; therefore, the prospect of a more appealing physical appearance may be a powerful motivator for behaviour change [10]. The aim of this study was to evaluate the effect of a personalised future self-image on weight change over a 6-month period, with a broader sample than the pilot study and to include both men and women of any age over 16 years.

## Methods

**Fig. 1:**
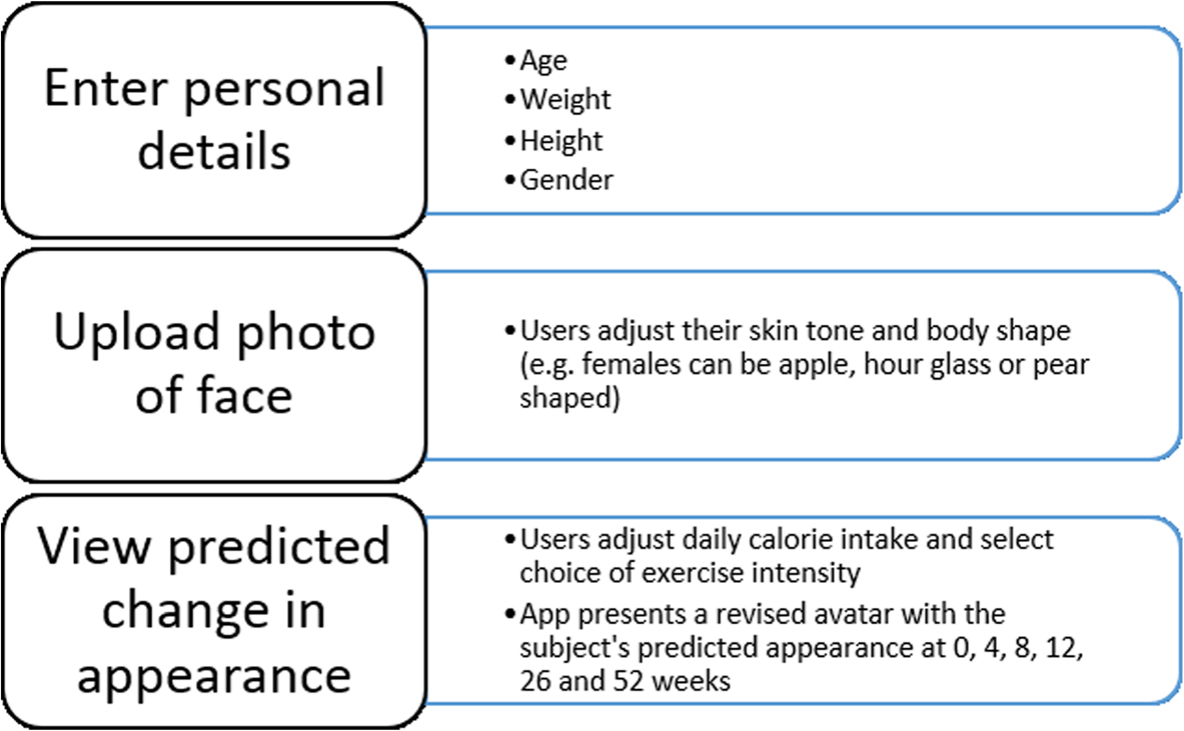
Features of the Future Me App (minimum viable product: MVP)

A computerised application (app) prototype called ‘Future Me’ was developed previously by the research team. The app portrays the effect of lifestyle on future personal appearance using input calorie and exercise information to predict future Body Mass Index (BMI) [9]. The minimum viable product (MVP) features are shown in Fig. 1 below.

The trial was approved by the Curtin Human Research Ethics Committee (HR 112/2013). A sample size of 150 was determined to have the power to detect a 1-kg weight difference between groups, which was the mean weight difference seen at 8 weeks in the pilot study [9]. A total of 145 participants, slightly fewer than the target number, were recruited over 8 months from November 2013 to August 2014, when the recruitment phase period concluded. Eligibility criteria required participants to be at least 16 years old, with a BMI over 25.0 kg/m^2^ and wanting to lose weight. Women who were pregnant or breastfeeding were ineligible. One participant was excluded due to recent thyroid surgery. During the study, one participant was withdrawn after becoming pregnant and another after opting for bariatric surgery. Participants were recruited through the Curtin University website, radio announcements, emails, and flyers. Recruitment took place at two general practices north and south of the Perth area, Curtin University, and two large employer groups located in the Perth metropolitan area.

At recruitment, participants completed a questionnaire outlining demographic details and an assessment of motivational state using the Prochaska Transtheoretical Model of Behaviour Change [11]. This model of behaviour change assesses an individual’s readiness to act on a new behaviour which can range from Pre-contemplation to Maintenance. In general the earlier the ‘stage of change’ for a behaviour the less readily an individual will act on a stimulus to change that behaviour.

Baseline height, weight, and waist circumference were measured. Then, all recruits were randomised, by selecting a concealed token produced beforehand using a random number generator, to receive a future self-image immediately (early-image group), or to receive the image after 8 weeks (delayed-image group). Based on the pilot study, 8 weeks was chosen as a reasonable amount of time for a difference in weight loss to emerge, and to retain sufficient numbers in the delayed-image group, whose members may be dissuaded by the need to return for weigh-ins after being denied the intervention at recruitment. To use the Future Me app, an iPad mini with the MVP installed was provided only during the meeting and operated by the participant with the researcher’s assistance. Each participant chose one preferred self-image from the set of five future time points at 4, 8, 12, 26, or 52 weeks. The current image (week 0) and the chosen future image were printed onto photo cards for the participant to keep.

Regardless of group allocation, all participants received 15 min of general lifestyle advice for weight loss. This advice included the following: to reduce overall calorie intake and/or increase physical activity in line with current guidelines [12]. To follow the National Health and Medical Research Council ‘Australian Guide to Healthy Eating’ chart [13]. Participants received a resource pamphlet listing several freely available online resources for weight management. It also listed a variety of professionals who could assist with weight loss, including accredited dieticians and accredited commercial weight-loss programmes (see Additional file 1).

**Fig. 2:**
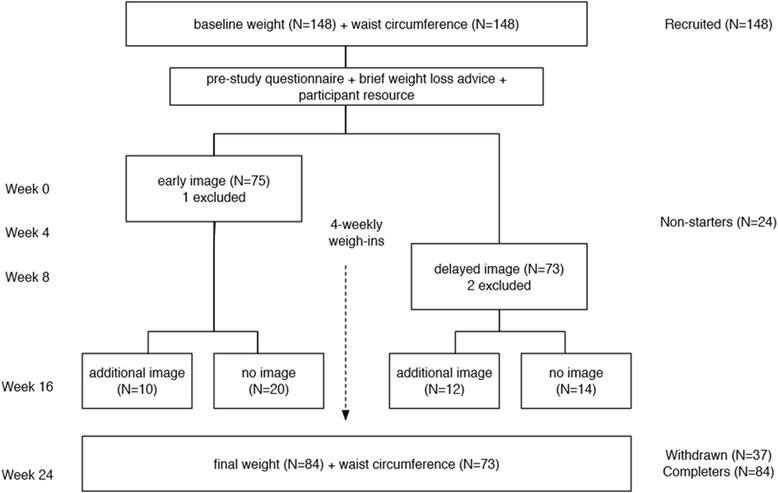
Consolidated Standards of Reporting Trials (CONSORT) diagram for the study. The numbers of participants at each stage are shown in brackets

Any weight-loss methods chosen were self-selected by the individual. Participants were asked to return once every 4 weeks for 24 weeks to record a weight measurement. The 4-week interval was chosen to minimise the impost on participants but to maintain close monitoring of their progress. Only weights recorded on the original set of scales or calibrated scales at alternative study locations were included in the final analysis. The researcher provided information on sources of advice at the time of weigh-ins, but did not provide any support in the intervening periods. At the 16-week visit participants were randomised again to either receive a second future self-image using their new weight parameters, or to continue with only the original future self-image. This step was designed to allow an estimate of the effect of repeated exposure to the image-creation process. Figure 2 summarises the study timeline.

The status of the participants was defined in three groups. Participants were deemed to have engaged in the study if they returned for the first weigh-in after recruitment. Participants who did not return for this visit are termed non-starters and were excluded from the analysis. Participants were deemed to have completed the study if they recorded a weight at either week 20 or week 24. Participants who discontinued weight measurements between week 4 and week 20 are termed dropouts and are included in the intention-to-treat (ITT) analysis.

### Missing data

It was common for participants to have an incomplete set of weight measurements. For completers, if a weight was recorded at 24 weeks, then any missing intermediate weight measures were substituted with a value obtained by linear interpolation from baseline to the 24-week observation. For example, if a subject was missing data at weeks 12 and 16, these values were estimated from the straight line joining values measured at baseline and at 24 weeks. In the three instances where a 20-week weight was available, but the final 24-week value was missing, the 24-week value was assumed to be the same as the 20-week value. For dropouts or withdrawals, a ‘last observation carried forward’ strategy was used to estimate all missing measurements, whereby each missing value was replaced by the weight at the previous time period.

### Data analysis

Standard descriptive statistics (frequencies and percentages for categorical variables, means, and standard deviations for variables measured on a continuous scale) were used to summarise the profile of participants in the study. Comparisons between treatment groups for these variables were performed using the chi-square test or *t* test as appropriate, to check whether the groups were similar at baseline.

The primary outcome was weight loss over 16 weeks. A ‘per-protocol’ analysis was performed using participants whose weights were measured at weeks 8 and 16 (at least). Attendance at week 8 was required for participants in the delayed-image group to receive their image, and the week 16 measurement was required for the final measurement (following protocol). A separate ITT analysis was also performed up to week 16 (with missing data replaced as described above).

Secondary outcomes included weight loss and change in waist circumference over 24 weeks. These analyses were undertaken firstly using only ‘completers’ who attended at weeks 8 and 16 (per protocol), and repeated including withdrawals (ITT). Analyses on weight were performed using a random effects regression model. The independent variables included in the model were time and treatment (delayed versus early image), and their interaction. The purpose of the interaction term was to identify whether the rate of weight loss over time differed between treatments. Subsequently, the model was expanded to identify whether weight loss also depended on any of the other factors measured. These variables included: recruitment location; gender; age group (18–35, 36–55, 56–79 years); marital status; following a weight-loss programme at baseline; and motivational stage (Pre-contemplation, Contemplation, Action, Maintenance). Analysis of the data to 24 weeks included a term indicating the group to which participants were allocated at 16 weeks. A backwards elimination strategy was used to identify the best model including these other factors. This was done by including all independent variables in the model at first, and then dropping them, one at a time, until all variables remaining in the model were significantly associated with weight change. At that point, all the pairwise interaction terms were assessed for significance. Change in waist circumference was the length at week 24 minus the length at baseline, and analysed using a paired *t* test.

The variable for time was initially included as a categorical variable, so that no assumption was made concerning the linearity of the change in weight over time. However, change in weight was close to linear, and so the variable was subsequently treated as continuous. The changes in weight are expressed as a percentage of baseline weight per 4-week interval (approximately ‘per month’).

Statistical analyses were performed using the SAS version 9.2 software, and, following convention, a *p* value < 0.05 was taken to indicate a statistically significant association in all tests.

## Results

### Group characteristics

One hundred and forty-five participants were recruited to the study, with 74 allocated to receive their image at commencement, and 71 to the delayed-image group. Baseline characteristics according to initial group allocation are presented in Table 1. Twenty-four recruits were deemed non-starters, comprising 15 (21%) in the delayed-image group and 9 (10%) in the early-image group (χ^2^_(1)_ = 2.1, *p* = 0.1465). Sixteen participants allocated to the early-image group, and 21 participants allocated to the delayed-image group withdrew, so that 84 (69.4%) of the 121 participants who engaged in the study, completed it. The completion rate was similar for early- and delayed-image groups (χ^2^_(1)_ = 2.35; *p* = 0.13) and across different categories of baseline motivational status (χ^2^_(2)_ =0.42; *p* = 0.8108).

**Table 1:** Baseline characteristics of participants recruited to the study. *p* values are obtained from the chi-square test, unless otherwise specified

Variable	Early-image *n* (%)	Delayed-image *n* (%)	*p* value
Participation status			0.11
Non-starter	9 (12.2)	15 (21.1)	
Withdrawal	16 (21.6)	21 (29.6)	
Completer	49 (66.2)	35 (49.3)	
Gender			0.34
Male	16 (21.6)	11 (15.5)	
Female	58 (78.4)	60 (84.5)	
Age group (years)			0.57
18 − 35	21 (28.4)	21 (29.6)	
36 − 55	38 (51.4)	31 (43.7)	
56 − 79	15 (20.3)	19 (26.8)	
Education			0.56
Tertiary	51 (68.9)	50 (70.4)	
Secondary	23 (31.1)	20 (28.2)	
Income			0.24
<AU$40 K	13 (17.6)	11 (15.5)	
AU$40 − 80 K	16 (21.6)	9 (12.7)	
AU$80 − 120 K	16 (21.6)	14 (19.7)	
AU$120 − 160 K	11 (14.9)	20 (28.2)	
> AU$160 K	14 (18.9)	16 (22.5)	
Partnered	51 (68.9)	49 (69.0)	0.99
Current programme	10 (13.5)	7 (9.9)	0.49
SOC			0.03
Contemplation	8 (10.8)	20 (28.2)	
Action	47 (63.5)	37 (52.1)	
Maintenance	19 (25.7)	14 (19.7)	
Weight-loss attempts			0.35
None	4 (5.4)	5 (7.0)	
1 or more	68 (91.9)	66 (93.0)	
BMI >30	47 (63.5)	36 (50.7)	0.12
Height (mean [SD])	166.2 (8.5)	165.6 (8.0)	0.75^a^
BMI (mean [SD])	33.5 (7.6)	32.2 (6.1)	0.20^a^
Waist (mean [SD])	101.0 (14.3)	98.5 (13.8)	0.21^a^

^a^Wilcoxon two-sample test. *BMI* Body Mass Index, *SD* standard deviation, *SOC* Stage of change

### Outcomes

#### Per-protocol analysis up to week 16

Of the 121 participants who engaged in the study, 55 attended at weeks 8 and 16 (the ‘per-protocol’ participants). Of these, 30 (46.2%) were in the early-image group and 25 (44.6%) were in the delayed-image group. The analysis showed a significant decline in weight over time (*p* < 0.0001), and also a significant difference in rate of change between early and delayed images (delayed-image: −0.77%, early-image: −0.49%; *p* = 0.018).

#### ITT analysis up to week 16

When the same model was applied to the ITT dataset, there remained a significant change over time (*p* < 0.0001), and a significant difference between groups (delayed-image: −0.50%, early-image: −0.30%, *p* = 0.007). This difference is smaller due to the missing value replacements that were performed in both groups.

#### Per-protocol analysis up to week 24

Similarly to the situation at 16 weeks, there appeared to be a very significant change in weight overall (*p* < 0.0001), and a difference in rate of change between groups (delayed-image: −0.60%, early-image: −0.42%, *p* = 0.012). The group into which participants were allocated at week 16 (second image or not) appeared not to influence the outcome (*p* = 0.3128).

#### ITT analysis up to week 24

Analysis of all completers and withdrawals showed a strong trend over time (*p* < 0.0001), and a difference in rate of change between groups (delayed-image: −0.50%, early-image: −0.27%, *p* = 0.0008).

Table 2 shows the results of analysis of the ITT dataset to week 24 (all records except non-responders), when other variables were included as candidate-independent variables. The final model included only gender (men lost weight at a greater rate than women), and treatment group (greater weight loss for the delayed-image group). The figures in the table show the overall relative weight loss per 4 weeks (−0.7%), and the differences in weight loss between genders and treatment groups. For example, the weight loss for men in the delayed-image group is estimated to be −0.7%/4 weeks. For women in this group, it would be −0.35% (−0.7 + 0.35). For those in the early-image group, the corresponding figures would be −0.49% for men, and −0.14% for women.

**Table 2:** Analysis based on all completers and withdrawals, up to week 24 (intention-to-treat (ITT) analysis; *N* = 121)

	Rate of change (% of baseline/4 weeks)	95% confidence interval	*p* value
Time	−0.70	−0.84 to −0.56	<0.0001
Gender			
Female	0.35	0.22 to 0.49	< 0.0001
Male	0.0 (reference)		
Treatment group			
Early-image	0.21	0.11 to 0.32	< 0.0001
Delayed-image	0.0 (reference)		

Mean change in waist circumference over 24 weeks was –2.74 cm for the delayed-image group and –3.23 cm for the early-image group, which was not statistically significant (*p* = 0.68). By week 24, 8 (12.3%) of the early-image and 12 (21.4%) of the late-image participants who were engaged in the study (ITT) achieved a weight loss of at least 5%. This difference was not statistically significant (*p* = 0.1780). When based on ‘completers’, the percentages rise to 14.3% of the early-image group and 28.6% of the delayed-image group (*p* = 0.1081).

## Discussion

This study has examined the rate of weight loss in adults who received a personalised image of predicted future body shape, with self-selected diet and exercise targets. On average, weight loss was modest over the 24-week period, but greater than has been recorded in other studies that have provided advice alone [14]. In this trial more than one fifth of completers in the delayed-image group lost 5% or more of their baseline weight.

A greater rate of weight loss was seen with the delayed-image group completers. This was an unexpected result. It was hypothesised that a longer period of exposure to the future self-image would result in more weight loss, favouring the early-image group. However, it may be that creation of the future self-image several weeks into a weight-loss attempt served as a greater trigger or additional reinforcement for further weight loss. An alternative explanation is that a greater number of participants in the delayed group were committed to weight-loss efforts. There was some evidence for this as a greater proportion of those in the delayed group were in the Contemplation phase of the change and a higher proportion were non-starters in this group. Failing to receive an early image may have prompted some recruits, those less committed to weight loss, to drop out early, leaving in comparison, relatively more committed recruits in the delayed-image group. We found, however, no significant interaction between initial motivational stage and percentage weight loss, and no significant effect of group allocation on continuation status, so this explanation is less plausible. Average age and baseline BMI were similar between the groups. Any apparent differences in male-to-female ratio and motivational stage would have in fact favoured weight loss in the early-image group.

Data from this study consistently demonstrated more rapid weight loss in men. Without information on individual dietary and physical activity records during the study it is not possible to confirm why this was the case, although the same pattern has been reported elsewhere [15]. Patterns of dietary change after weight-loss advice have been described in the medical literature for both men and women [16, 17]. It may be that the male participants made more effective lifestyle changes. Weight loss for men and women can be expected to be similar when energy expenditure is also comparable [18]. The difference favouring more rapid weight loss in the delayed-image group was observed for men in the ITT analysis. The reason is unclear, but suggests that there may be a disparity between genders in how the image operates.

We found no significant difference between groups for weight loss at the 8-week weigh-in where the delayed group had not yet viewed the future self-image. Comparable results between groups at 8 weeks may be because it was not a long enough period of time to produce a divergence or, as already postulated, the effect of the future self-image was less significant at the outset of a weight-loss attempt.

Visual appearance is highly important for most people who want to lose weight [10, 19]. The Future Me app allows the user to actually visualise the changes that they could achieve through lifestyle modification. Furthermore, the user is provided with an estimate of the effort in terms of diet and exercise, as well as the time required to achieve, the desired physical appearance. These factors are likely to help set realistic goals. The Future Me images can be created within minutes using only basic nutritional knowledge. These features make it an ideal tool for time-constrained, non-specialist clinical settings such as general practice.

The myriad health benefits achieved with weight loss for those in the overweight and obese range are well established. The use of a future self-image does not imply that the health message is any less important than physical appearance. Rather, the method acknowledges how important physical appearance is for many dieters, and allows a tangible visualisation of healthy lifestyle change.

## Strengths and limitations

This study reports a randomised trial of an iPad app. The subjects were followed up for 24 weeks and the data are reported on an ITT basis. The team did not feel that any group could be randomised to have no access to the app because communication between participants may have led to disappointment and frustration for those participants who would have not received it. Nonetheless, we acknowledge that this reduces our capacity to assess the impact of the app against no intervention. Also, the attempt to test the dose effect of the app at 16 weeks was unsuccessful due to low attendance at the 16-week weigh-in where only 22 participants were allocated a second future self-image. In general, there was significant attrition during the study as appears to be case for many studies reporting weight-loss interventions. Sixteen percent of recruits were non-starters and the attrition rate for the remainder was 31%. This is on par with most weight-loss trials where attrition is commonly between 20 and 50%, and sometimes more [20].

## Conclusion

A personalised future self-image may boost weight-loss efforts when implemented in the course of an ongoing weight-loss attempt. The Future Me app allows such images to be created in time-constrained clinical settings. Further research with this tool is needed to determine its overall efficacy and the optimal way to implement the app with existing weight management strategies.

## Additional file

Additional file 1: The Participant Resource Brochure. (PNG 340 kb)

## Abbreviations

App: Application
BMI: Body Mass Index
CI: Confidence interval
kg: Kilogram
MVP: Minimum viable product
