# Supplementary figures and images for: Do images of a personalised future body shape help with weight loss? A randomised controlled study
Source: Trials. 2017 Apr 18;18:180. doi: 10.1186/s13063-017-1907-6 (PMC5395810; doi:10.1186/s13063-017-1907-6)

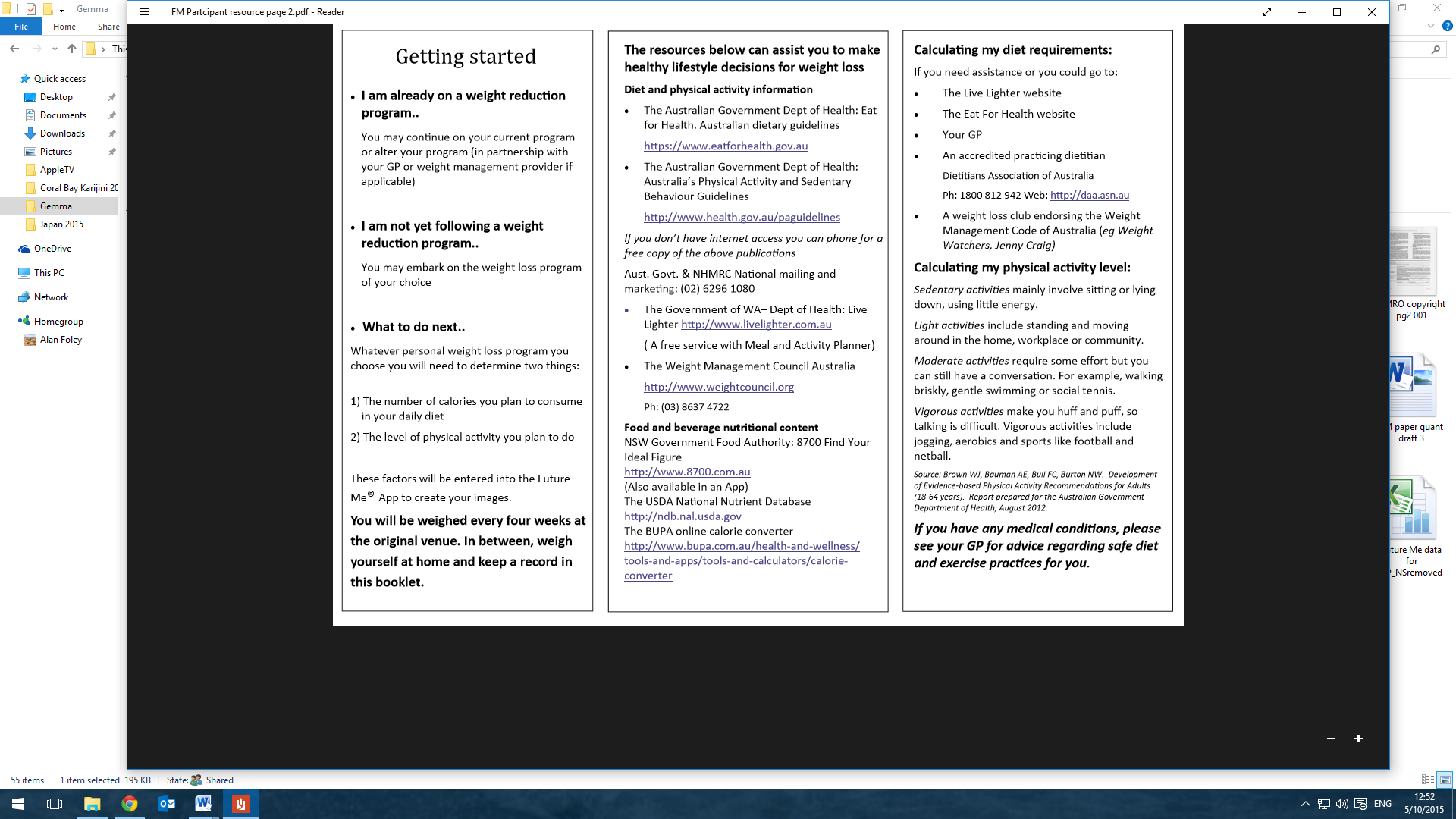

Supplement: Additional file 1: — The Participant Resource Brochure. (PNG 340 kb) [file 13063_2017_1907_MOESM1_ESM.png]
